# A Pharmacological Primer of Biased Agonism

**DOI:** 10.2174/187153011795564179

**Published:** 2011-06

**Authors:** Bradley T Andresen

**Affiliations:** 1 Department of Internal Medicine, Division of Endocrinology, University of Missouri; 2 Department of Medical Pharmacology and Physiology, University of Missouri; 3 Harry S Truman Veterans Affairs Medical Center, USA

**Keywords:** Arrestin, beta-adrenergic receptors, biased agonism, G protein-coupled receptors (GPCRs), G protein-coupled receptor kinases (GRKs), pharmacology.

## Abstract

Biased agonism is one of the fastest growing topics in G protein-coupled receptor pharmacology; moreover, biased agonists are used in the clinic today: carvedilol (Coreg^®^) is a biased agonist of beta-adrenergic receptors. However, there is a general lack of understanding of biased agonism when compared to traditional pharmacological terminology. Therefore, this review is designed to provide a basic introduction to classical pharmacology as well as G protein-coupled receptor signal transduction in order to clearly explain biased agonism for the non-scientist clinician and pharmacist. Special emphasis is placed on biased agonists of the beta-adrenergic receptors, as these drugs are highly prescribed, and a hypothetical scenario based on current clinical practices and proposed mechanisms for treating disease is discussed in order to demonstrate the need for a more thorough understanding of biased agonism in clinical settings. Since biased agonism provides a novel mechanism for treating disease, greater emphasis is being placed to develop biased agonists; therefore, it is important for biased agonism to be understood in equal measure of traditional pharmacological concepts. This review, along with many others, can be used to teach the basic concepts of biased agonism, and this review also serves to introduce the subsequent reviews that examine, in more depth, the relevance of biased agonism towards the angiotensin type 1 receptor, parathyroid hormone receptor, and natural biased ligands towards chemokine receptors.

## INTORDUCTION

Biased agonism, also known as functional selectivity, is a rather recent discovery regarding the fundamental nature of how G protein-coupled receptors function. Put simply, biased agonism refers to a ligand’s ability to signal through a subset of the classical signaling pathways initiated by the cognate receptor. Importantly, at least one biased agonist is in widespread clinical use: carvedilol (Coreg CR^®^) [1]. Therefore, it is important for clinicians, pharmacists, and educators to have a general understanding of biased agonism. The goal of this review is to: 1) describe biased agonism in the context of general pharmacological principles, 2) introduce the biased agonists of beta-adrenergic receptors (β-ARs), 3) discuss possible clinical benefits and complications of these compounds, and 4) introduce the proceeding detailed reviews about specific biased agonists. 

## CLASSICAL PHARMACOLOGICAL DEFINITIONS OF LIGANDS AND LIGAND EFFECTS

The following is a list of terms that are commonly used in pharmacology, but when dealing with biased agonism of antagonists it is wise to revisit the original definition and how these definitions have “evolved” as we have become more aware of the complexities of receptor ligand interactions and the subsequent biological effect of said interaction. For a more detailed and thorough review of these topics please see the following excellent reviews by Terry Kenakin [2-5].

### Ligand

– a molecule that binds to a receptor.

### Potency

**Fig. (1):**
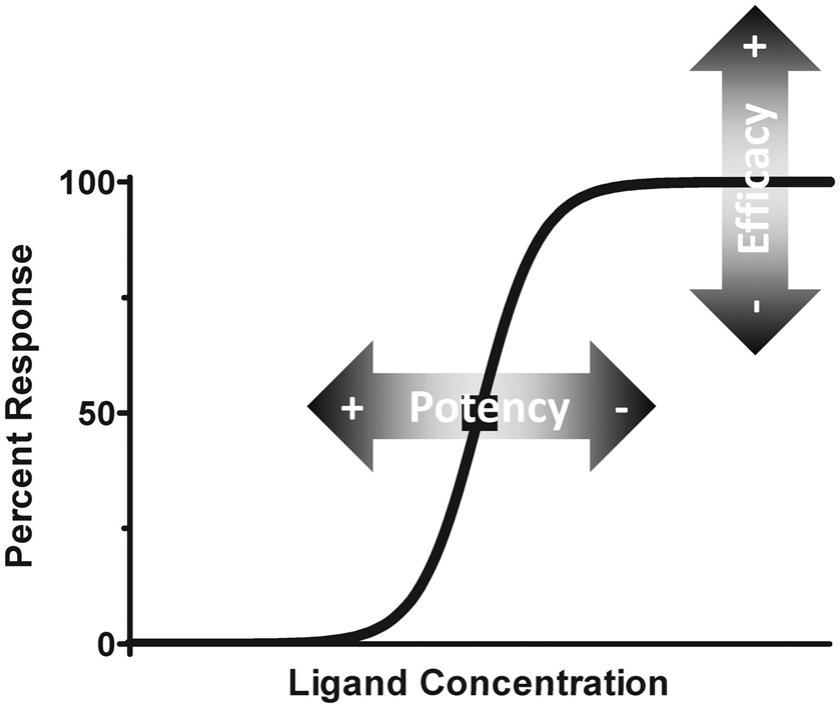
Depiction of classical pharmacological terminology. Depicted is a typical pharmacological dose-response curve using a linear Y-axis and a Log_10_ X-axis; the curve that is generated is a sigmoidal curve. The midpoint of the curve (50% response represented by ■) is the effective concentration 50 (EC_50_), which is used to rank agonists. A compound can have greater or lesser efficacy and potency (shown as shaded arrows).

– the measurement of the concentration of a ligand in respect to a biological response (Fig. **1**). Potency is generally denoted by an EC_50_ value, which indicates the ligand concentration required to reach half maximal effect.

### Efficacy

– the measurement of the maximal response of a ligand (Fig. **1**). 100% efficacy is generally defined by the endogenous agonist, and represents the upper asymptote of a sigmoidal curve.

### Agonist

– a molecule targeting a receptor that elicits a biological response; traditionally this response was similar to the endogenous ligand’s effects. However, an agonist can have either increased or decreased potency and/or efficacy. Generally, an agonist with markedly reduced efficacy is termed a partial agonist for it has partial effects.

### Antagonist

– a molecule that blocks the action of the agonist and endogenous ligand. A classical example is the competitive antagonist which has no effect on the target receptor, yet binds to the receptor blocking an agonist from binding to the receptor and eliciting a biological effect. In Fig. (**1**), the addition of a competitive antagonist would shift the curve to the right; greater ligand concentration would be required to displace the antagonist and result in a biological effect.

The above definitions have been challenged previously and thus new ideas have entered into the classical pharmacological lexicon; two instances deserve discussion: inverse agonism and selective receptor modulators. Inverse agonism, first described in 1982 [6], indicates that a ligand will produce negative efficacy. This phenomenon is attributed to receptors that display basal activity that is concentration-dependently inhibited to an asymptote below the basal activity; ideally this would be towards no activity (an asymptote of 0). If this were to be depicted in Fig. (**1**), the sigmoidal curve would start at a positive integer (25 for example) and the inverse agonist would reduce the activity following a sigmoidal curve (from 25 to 0, for example). As with any agonist, the potency and efficacy could vary. Selective receptor modulators, first described in 1987 [7], are ligands that behave differently depending on the tissue being studied; they can be an agonist in one tissue and simultaneously an antagonist in another. Prototypical examples of selective receptor modulator are the selective estrogen receptor modulators (SERMs). The mechanism underlying the paradoxical action of SERMs is the tissue specific expression of co-activators and co-repressors of the estrogen receptors that are required for the ligand to act as an agonist or antagonist [8]. These early challenges to classical concepts of pharmacological effects foreshadowed the growing complexities of ligand-receptor interactions and cellular signaling that are still being unmasked. Currently the definitions of the potential pharmacological action of a ligand are complex and can be defined observationally and functionally (Table **1**).

## CLASSICAL GPCR SIGNALING AND REGULATION

Thus far, only G protein-coupled receptors (GPCRs) are reported to have biased agonists, and to understand biased agonism a basic understanding of GPCR-mediated signaling is required. Since GPCRs are the largest single grouping of receptors and represent the largest class of clinical drug targets [9], biased agonism and traditional agonists and antagonists will play a role in the treatment of a variety of diseases.

**Fig. (2):**
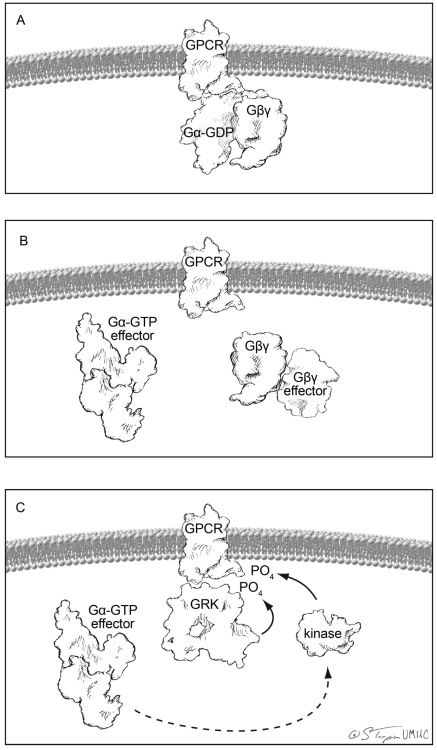
Depiction of classical GPCR initiated signaling and desensitization. All cartoons were created from published crystal structures. (**A**) Basal state of a GPCR docked to the heterotrimeric Gprotein. (**B**) Canonical ligand initiated signaling separates and the Gα and Gβγ subunits from each other and the receptor allowing for each to initiate signal transduction cascades. (**C**) Homologous desensitization is initiated by GRK-dependent phosphorylation of the receptor and signaling-induced phosphorylation of the receptor by PKA or PKC.

GPCRs are proteins that cross the plasma membrane seven times; their amino terminus is outside of the cell and the carboxy terminus within the cytoplasm. A GPCR is coupled to an inactive GDP-bound Gα subunit and a Gβγ dimer (Fig. **2A**). There are multiple Gα, Gβ, and Gγ subunits, which will not be discussed in detail in this review. Traditionally, an agonist binds to the receptor which initiates guanylyl exchange on the Gα subunit (GDP is exchanged for GTP), which places Gα into an active state. The active Gα and Gβγ then leave the receptor and bind to their respective targets in the cell, thus propagating the signal and initiating the cellular signaling cascade from the GPCR (Fig. **2B**). This initiation is the beginning of cellular signaling and is typified by, but not limited to, activation (Gα_s_) or inhibition (Gα_i_) of adenyl cyclase production of cAMP, activation of phospholipase C (Gα_q/11_) and RhoA (Gα_12/13_).

Shortly after activation of the GPCR, the GPCR is phosphorylated by a number of kinases (Fig. **2C**). These phosphorylation events can be classified into two different varieties: signaling-mediated and signaling-independent GPCR phosphorylation. Signaling-mediated GPCR phosphorylation is mediated by kinases that are directly activated by the GPCR signaling cascade such as PKA [10], which is activated by increased levels of cAMP, and PKC [11], which is activated by multiple signaling pathways but is classically downstream of phospholipase C. Importantly, these kinases, once activated by a GPCR, can phosphorylate other GPCRs; a process called heterologous desensitization [12]. Signaling-independent GPCR phosphorylation occurs through GPCR kinases (GRKs), which have been shown to phosphorylate GPCRs that are in an active state, such as when a GPCR is bound to an agonist [13, 14]. Phosphorylation of GPCRs initiates the desensitization process [15] through recruitment of β-arrestins [16]. β-arrestins are scaffolding proteins that bind to the phosphorylated receptor [17] and are involved in clathrin-mediated GPCR internalization [18].

In summary, basic GPCR action relies on an agonist to activate the Gα and Gβγ subunits to initiate signaling and then the GPCR is phosphorylated/desensitized and internalized. At least that was the view of GPCR-mediated signaling for many years until it was realized that β-arrestins are signaling molecules in their own right [19]. Therefore, the desensitization process initiated by phosphorylation activates unique cellular signaling pathways. Consequently GRKs, which can phosphorylate a GPCR in a signaling-independent fashion, are involved in signal propagation. This raises an interesting question: can the GRK/arrestin pathway be activated independently of classical GPCR-mediated (Gα and Gβγ) signaling? Indeed it can, as this is one form of biased agonism.

## BIASED AGONISM

Biased agonism refers to the ability of a ligand to activate a subset of a receptor’s signaling cascade. For GPCRs this subset is either the β-arrestin-mediated signaling events or Gα and Gβγ events but not both pathways simultaneously as would occur with a traditional agonist. Consequently, biased agonists that only activate the β-arrestin pathway appear to be antagonists if the only output that is examined is the Gα- and Gβγ-mediated events, which is the traditional method for classifying ligands. Consequently, the β-AR biased agonist carvedilol has been marketed as a β-AR antagonist because its β-arrestin activating pathway was not discovered until 2007 [1]. On the other hand, biased agonists that activate Gα and Gβγ have been classified as agonists, which is closer to their true identity than the previous example. Yet these agonists would be long acting for they would not recruit β-arrestins and thus escape the signaling-independent desensitization and internalization process.

GPCR-mediated signaling is dependent on the conformation (3-Dimensional sape) of the receptor [20]. Agonists shift the inactive (neutral) receptor conformation towards one that promotes signaling. Pure competitive receptor antagonists do not alter the conformation of the receptor yet prevent the binding of an agonist and therefore prevent agonists from shifting receptors to the active conformation. Inverse antagonists not only prevent the binding of agonists, but they also promote a shift of constitutively active receptors to the inactive conformation. Since the specificity of GRKs are believed mainly to be determined by the conformation of the GPCR [21, 22] and β-arrestins bind to phosphorylated receptors [16], this would indicate that most β-arrestin biased agonists place the receptor into a confirmation that is recognized by the GRK but not the Gαβγ heterotrimer allowing for phosphorylation and β-arrestin recruitment sans classical GPCR-mediated signaling. There is the possibility that a GPCR can interact directly with a β-arrestin independent of phosphorylation [23]; however, these studies were conducted solely *via *cellular assays with mutant receptors that cannot be phosphorylated, which indicates that this pathway is possible but may not occur in natural conditions. Supporting this, transgenic mice expressing a β1-AR that cannot be phosphorylated by GRKs develop more severe cardiac dysfunction after prolonged isoproterenol (a β-AR agonist) stimulation than mice expressing similar levels of wild-type β1-ARs [24]. Taken together, the biochemical properties of biased agonists can be thought of as a partial conformational change, or more accurately a limited regional shift in the shape of the GPCR [25]. All proteins are three dimensional structures; thus it is not hard to imagine that there could be a shift in one area of the structure while there is little to no change in a second area. Using this concept, a GPCR will have a number of possible shapes; as depicted in the Venn diagram of Fig. (**3**), some shapes will initiate Gα and Gβγ as well as GRK/β-arrestin signaling cascades (traditional agonists) while other shapes only initiate Gα and Gβγ or GRK/β-arrestin signaling cascades (biased agonists) or confer no signal transduction (pure competitive antagonists and inverse agonists).

The biochemical basis of this shape change is actively being examined; however, some mechanisms can be deduced from the current literature. Gα proteins interact with the receptor in a small pocket near the membrane, and GRKs interact with the same pocket, which is enlarged when an agonist is bound to the receptor [22]. Therefore, the Gα-Gβγ heterotrimer and GRK cannot simultaneously interact with the GPCR due to steric hindrance. Thus, biased agonists must either activate undocked receptors (GPCRs that are not bound to G proteins) or the conformation induced by the biased agonist passively displaces the Gα-Gβγ heterotrimer from the GPCR. In this instance “passively” indicates that the GPCR is not initiating guanylyl exchange on the Gα subunit, but is initiating a separation of the heterotrimer from the GPCR. Upon docking of the GRK to the GPCR, the receptor will be phosphorylated and β-arrestins will be recruited thus allowing for β-arrestin-mediated signaling.

## CARVEDILOL AND ALPRENOLOL

Carvedilol and alprenolol are now rather old β-adrenergic receptor antagonists, aka β-blockers, that have recently been shown to actually behave as biased agonists [1, 26]. Alprenolol was first published in a clinical study in 1968 [27] and is no longer actively marketed; whereas carvedilol is a third generation β-blocker, approved by the FDA in 1995, that has relatively recently (9/5/2007) been classified as a generic drug [28, 29]. Additionally, on 20 October 2006 the FDA approved a controlled release tablet of carvedilol from GlaxoSmithKline marketed as Coreg CR^®^ for the treatment of heart failure, hypertension, and post-myocardial infarction left ventricular dysfunction [29]. The total combined sales in 2009 of carvedilol (Coreg CR^®^ and generic carvedilol) are over 0.5 billion dollars, which represents over 17 million prescriptions [30]. Therefore, there are many people being treated with a biased agonist, and since carvedilol is a well tolerated β-blocker [31] it will likely be among the first line choices for many physicians prescribing generic β-blockers. Furthermore, there are additional β-blockers that have not been tested for biased agonism, and there are likely to be β-blockers that are formulated in the future, thus there may be more clinically viable β-blockers that are also biased agonists than just carvedilol.

As a biased agonist carvedilol and alprenolol bind to β-ARs and prevent the endogenous agonists, norepinephrine and epinephrine, from binding to the receptor and initiating full receptor-mediated signal transduction. Since the β-ARs predominately active Gα_s_-mediated production of cAMP, monitoring production of cAMP or GTP-bound Gα_s_ was used, along with binding studies, to show that carvedilol and alprenolol are β-blockers. In retrospect, these experiments were not sufficient to fully characterize carvedilol and alprenolol as the GRK/β-arrestin pathway was ignored. The full extent of β-AR initiated GRK/β-arrestin signaling is actively being explored; however, it is clear that both carvedilol and alprenolol lead to activation of the epidermal growth factor receptor (EGFR) through β-arrestins [26]. 

The EGFR is infamous for its role in cancer [32-34]; however, it is also involved in regulation of nitric oxide (NO) production [35, 36], and has been shown to induce relaxation of various vascular beds [37, 38]. Consequently, it is not surprising that carvedilol has been shown to induce NO production [39] and that a significant portion of its antihypertensive properties are dependent on NO production [40]. Moreover, NO and drugs that enhance NO effects are used as a treatment for a variety of cardiovascular diseases [41]; therefore, it is likely that many of the beneficial properties that make carvedilol unique compared to other β-blockers are due to carvedilol’s biased agonism properties.

## POTENTIAL RISKS OF PRESCRIBING A β-AR BIASED AGONIST

Foremost, there is no evidence that β-AR biased agonists cause cancer. Thus far antihypertensive drugs, as a class, have been shown to have little to no effect on a patient’s risk of developing cancer [42-44]; however, no clinical or basic studies have been conducted specifically with carvedilol. Anecdotally, biased agonists have been prescribed for more than 15 years and there is no report that a biased agonist β-blocker is linked to cancer. Additionally, stimulation of the EGFR by a GRK/β-arrestin pathway is not identical to activating EGFR directly [45, 46], and there is a report indicating that carvedilol inhibits multiple cancer cell lines growth *in vitro* [47]. Therefore, activation of the EGFR by carvedilol is a greater good due to the role of NO in the cardiovascular system than the hypothetical risk of inducing a cancer. But, there is a select group of patients that pose an interesting scenario for why understanding and classifying antagonists as biased agonists versus traditional antagonists is important for physicians and pharmacists.

Within the past decade multiple cellular and animal models have demonstrated that β-ARs are involved in multiple types of tumor proliferation [48, 49], invasion [50-53], and *in vivo* metastasis [54, 55]; moreover β-ARs are present on many human tumors [53, 55]. In all cases where β-blockers (generally propranolol) were used, it was found that blocking β-ARs reduces tumor proliferation, invasiveness, and metastasis suggesting that β-blockers can be used as antineoplastic drugs. This is supported by two lines of clinical evidence. First β-blockers reduce the risk of a patient developing cancer [44, 56]. Second, propranolol (a β-blocker) is considered a first line of therapy for infantile hemangiomas, which is benign tumor-like malformation, due to propranolol-induced rapid involution of the hemangioma [57]. Therefore it is reasonable to prescribe a β-blocker to cancer patients; however, as the EGFR is involved in many cancers [32-34] care should be used when prescribing a drug that activates the EGFR, such as biased agonists.

To demonstrate this concern a closer look at β-ARs in breast cancer serves as an interesting example. Most breast cancers express β-ARs [55, 58], and there is a striking correlation between EGFR levels and β2-AR levels [58]. Furthermore, in a breast cancer cell line (MCF-7) that is routinely used as a cellular model of breast cancer, β-ARs stimulate the production of EGFRs, and EGF leads to the synthesis of the catecholamine biogenesis pathway and increased levels of epinephrine [58]. Therefore, a vicious circle is formed where the EGFRs, which are a target of the anti-neoplastic agent Herceptin®, are producing agonists to the β-ARs, which are involved in breast cancer metastasis [54]. Completing the circle, β-AR activation leads to the expression of more EGFRs, which could conceivably lead to acquired resistance to EGFR inhibitors resulting in the need to prescribe higher doses of the drug. Thus, treating breast cancer patients with an EGFR inhibitor and β-blocker is recommended because the β-blocker can reduce tumor proliferation and metastasis [49, 54]; thus, increasing the probability of a optimistic prognosis. Yet, treatment with a biased agonist would activate the EGFR, which may be counterproductive. 

Thus far no *in vivo* tests or human trials have been conducted to examine the role of carvedilol in breast cancer, and as stated previously carvedilol may be a viable anti-neoplastic agent specifically for breast cancer [47]. However, since EGFR expression is associated with decreased survival of breast cancer patients [59], activating the EGFR through a biased agonist may not be beneficial even if the patient is taking an EGFR inhibitor. This is due to there being a greater likelihood that the EGFR would be activated as the concentration of the inhibitor decreases (normal clearance and biodistribution) or if the patient fails to take their medication as prescribed. Consequently, the EGFR might be activated by the biased agonist. Additionally, if transactivation of the EGFR signals similarly to EGF-mediated activation of the receptor in breast tumors, biased agonists would produce epinephrine [58]. This would reduce the clinical efficacy of the β-blocker/biased agonist due to an increased local concentration of epinephrine and competition for binding sites, suggesting that the patient would acquire resistance to β-AR biased agonists. This resistance would indicate that the β-ARs are likely being activated, and if a biased agonists is used at a higher dosage this scenario could become repetitive. More studies should be conducted to determine the potential positive or negative roles of β-AR biased agonists, such as carvedilol, in breast cancer treatment. Until these studies are conducted, biased agonists that activate growth promoting pathways should be used with care in any patient that is currently diagnosed with any cancer. As stated previously, there is no evidence that these drugs cause cancer, but there is also no *in vivo* or clinical evidence to counter the hypothesis that they may promote an existing cancer. Therefore, in these cases a pure β-blocker may be more appropriate.

As biased agonism is a new concept, there are many clinicians, pharmacists, and educators that do not know the possible ramifications of these drugs. Therefore, it is important to identify biased agonists. Importantly, biased agonism is a great new concept that provides a revolution in drug development and treatment of a variety of diseases. Carvedilol demonstrates that biased agonism is clinically viable, safe, and can provide additional beneficial clinical characteristics compared to traditional antagonists.

## CONCLUSION

The preceding section demonstrates that there is a need for the medical community to understand and continue to study biased agonism; additionally, biased agonism is an important new concept in pharmacology that has clinical relevance today. Coreg CR^®^ and the generic carvedilol are currently prescribed to millions of people, and there are situations where a non-biased agonist β-blocker may be a preferred treatment. Biased agonists are most easily described as an agonist that activates only one portion of a receptor’s signaling pathway while inhibiting the other portion. Therefore, all GRK/β-arrestin biased agonists will appear to be antagonists when only examining G protein-mediated signaling, as is the case of most drug discovery programs. Due to the current clinical use of biased agonists, it is important for physicians and pharmacists to understand which drugs are biased agonists and in which cases they should be used and avoided. Educators too should begin teaching biased agonism alongside traditional pharmacological definitions thus allowing for at least one more definition to be added to the initial list.

Biased Agonist – a molecule that activates: 1) the GRK/β-arrestin signaling pathways of a GPCR independent of the Gαβγ trimer, or 2) the Gαβγ trimer signaling pathways of a GPCR independent of GRK/β-arrestin.^*^

## Figures and Tables

**Fig. (3):**
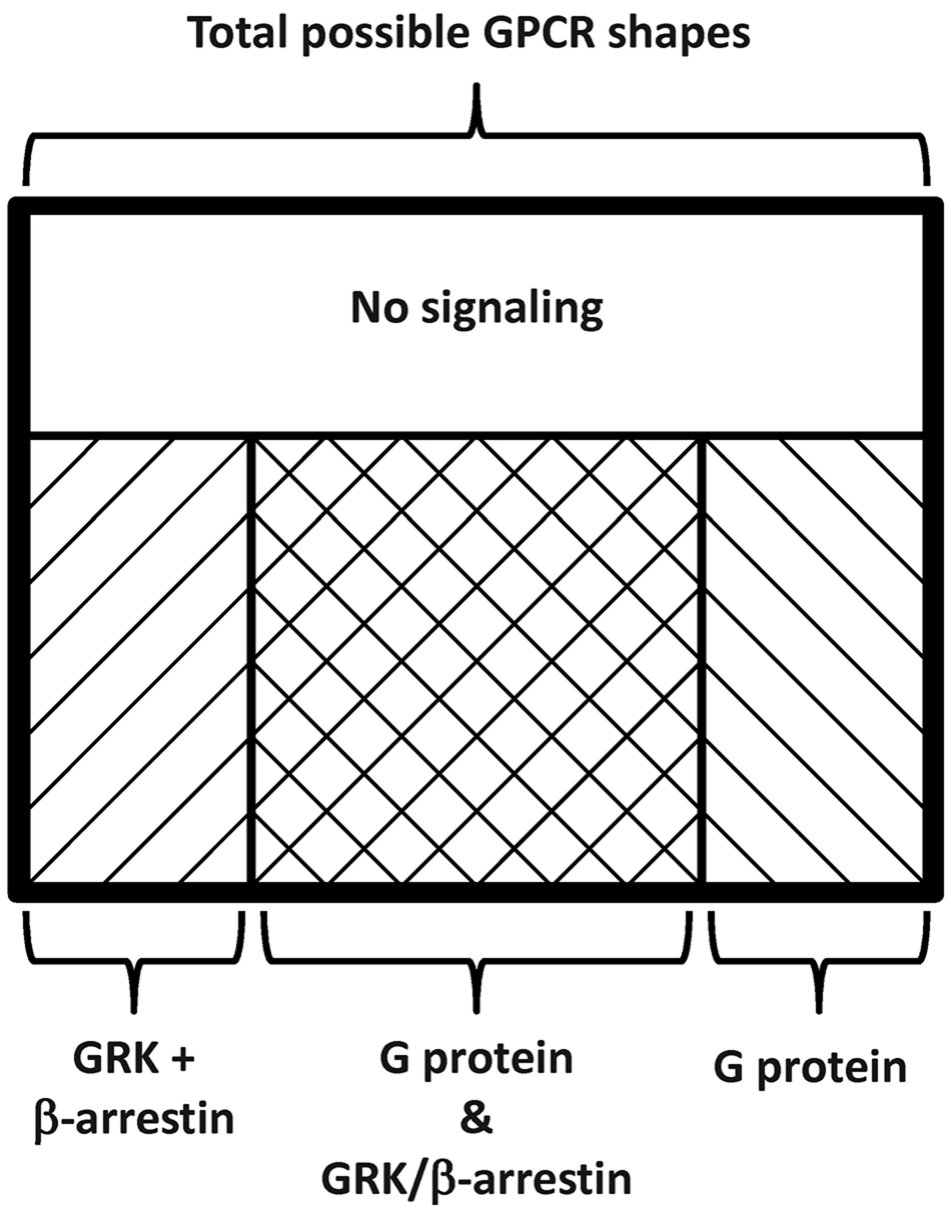
Modified Venn diagram representing the relationship between GPCR shape and signaling. Receptor shape dictates signaling, and there is only a certain range of shapes that can be formed by a functional GPCR (thick black box). Within those range of shapes some will resulted in no signaling (clear box), some in traditional signaling (cross hatched box), while others in biased agonism of the G-protein (rightward slant) or β-arrestins (leftward slant).

**Table 1:** Definitions of Ligands with Pharmacological Properties

Class	Type	Operational Definition	Molecular Mechanism(s)
Agonist	Full	Ligand generates maximum receptor-mediated responses of all responses linked to the receptor.	Ligand binds to the receptor and induces a receptor conformation that results in maximum signaling of all signaling pathways linked to the receptor.
	Partial	Ligand generates sub-maximum receptor-mediated responses of all responses linked to the receptor.	Ligand binds to the receptor and induces a receptor conformation that results in sub-maximum signaling of all signaling pathways linked to the receptor.
	Inverse	Ligand generates receptor-mediated opposite (or negative) responses compared to the full agonist.	Ligand binds to the receptor and induces a receptor conformation that results in no signaling by all signaling pathways linked to the receptor.
	Functionally Selective	Ligand generates receptor-mediated responses of a subset of responses linked to the receptor. A functionally selective agonist can be either full or partial.	Ligand binds to the receptor and induces a receptor conformation that unequally activates the signaling pathways linked to the receptor.
Selective Receptor Modulation		Ligand behaves as an agonist in some cells and as an antagonist in other cells.	Differentially expressed co-factors interact with the receptor and modulate the type of conformational change induced by the agonist
Antagonist	Competitive aka Surmountable	Ligand decreases the potency, but not efficacy, of an agonist (i.e., the full effect of the agonist can be restored at increased concentrations).	Several mechanisms can produce surmountable antagonism. Commonly, the underlying mechanism is competition between reversible antagonist and agonist binding at the receptor binding pocket and thus blocking the binding of the agonist. The phenomenon of surmountable antagonism, however, can also be produced by several types of allosteric (a site on a receptor that is different from the agonist binding site) mechanisms.
	Non-competitive aka Insurmountable	Ligand decreases the efficacy of an agonist, but may or may not reduce potency of an agonist (i.e., the full effect of the agonist cannot be restored at increased concentrations).	Several mechanisms can produce insurmountable antagonism. Common mechanisms include: 1) irreversible binding of the antagonist ligand to the binding pocket; 2) some types of allosteric mechanisms; and 3) interference of the antagonist with downstream signal transduction mechanisms linked to the receptor.

## ABBREVIATIONS

cAMP: = 3'-5'-cyclic adenosine monophosphate
EC_50_: = Effective concentration 50%
FDA: = United States Food and Drug Administration
GDP: = Guanosine-5'-diphosphate
GTP: = Guanosine-5'-triphosphate
PKA: = Protein kinase A
PKC: = Protein kinase C
